# Isolation, Characterization, and Pathogenicity of a Novel Recombinant Fowl Adenovirus 8a Strain in China

**DOI:** 10.3390/v18080847

**Published:** 2026-08-02

**Authors:** Aijing Liu, Chaohailan Wang, Jianing Chen, Enyan Meng, Xinru Zhao, Qian Zhang, Xiaojing Hao, Chunguo Liu, Qingqing Song, Zhaoyang Li, Gen Li, Qing Pan

**Affiliations:** 1 College of Veterinary Medicine, Qingdao Agricultural University, Qingdao 266109, China; laj_91@126.com (A.L.); akhlhlhl@163.com (C.W.); chenjn820@163.com (J.C.); enyanmeng@163.com (E.M.); zxruui@163.com (X.Z.); lizhaoyang@sinder.cn (Z.L.); 2 Dongying, Animal Health, Observation and Research Station, Ministry of Agriculture and Rural Affairs, Dongying 257000, China; 3 Qingdao Animal Husbandry Workstation, Qingdao 266100, China; 18661718134@163.com (Q.Z.); lovemenghaoyuan@163.com (X.H.); 4 Shandong SINDER Technology Co., Ltd., Qingdao 266104, China; liuchunguo@sinder.cn (C.L.); songqingqing@sinder.cn (Q.S.)

**Keywords:** FAdV-8a, complete genome, recombination analysis, pathogenicity, animal model

## Abstract

Inclusion body hepatitis (IBH), a disease predominantly associated with fowl adenovirus serotypes 8a (FAdV-8a) and 8b (FAdV-8b), poses a significant challenge to poultry production on a global scale. This study reports a severe outbreak of IBH that occurred in 2021 at a chicken layer farm in Hunan Province, China. Liver tissues were collected from visibly affected layer chickens for viral isolation in LMH cell cultures. Subsequent phylogenetic analysis was conducted based on DNA sequencing and bioinformatics data to determine the genetic relationships of the isolated virus. Pathogenicity trials were performed on specific-pathogen-free (SPF) chickens. A novel FAdV-8, designated HuN21, with recombination between serotypes FAdV-8a/FAdV-8b, was isolated. Recombination and phylogenetic analyses revealed that this recombinant strain harbored a genomic backbone and fiber gene from FAdV-8a and FAdV-8b, respectively. HuN21’s pathogenicity differed significantly by age groups, showing high pathogenicity (100% mortality, classic IBH signs) in 1-day-old SPF chickens, compared with no mortality despite viral replication in 28-day-old chickens. These data provide robust evidence for recombination between different serotypes of FAdV-8. Notably, the unique genetic and pathogenic characteristics of the HuN21 strain indicate potential as a candidate strain for the development of a bivalent inactivated vaccine or a natural vaccine against FAdV-8a/FAdV-8b.

## 1. Introduction

Fowl adenoviruses (FAdVs) [1] are viruses belonging to the family Adenoviridae described under the genus *Aviadenovirus*, classified as non-enveloped double-stranded DNA viruses with a genome of size of 43–45 kb [2,3]. FAdVs are divided into five species (FAdV-A-E) and 12 serotypes (FAdV-1–7, FAdV-8a, FAdV-8b, and FAdV-9–11) based on cross-neutralization experiments and restriction enzyme digestion patterns [3]. FAdV infection can cause various diseases, including gizzard erosion (GE), inclusion body hepatitis (IBH), and hepatitis-hydropericardium syndrome (HHS) [3,4]. IBH is primarily associated with fowl adenovirus serotypes 2, 11, 8a, and 8b [4,5], and is characterized by congestion and enlargement of the liver, accompanied by hepatic necrosis [6].

Since the widespread outbreak of HHS in Chinese chicken flocks in 2015, multiple FAdV serotypes have circulated in this region. The escalating dominance of FAdV-8a and FAdV-8b strains has created substantial obstacles for effective disease control protocols. Although FAdV-8 is less virulent than FAdV-4, and thus commonly overlooked, the pathogen often acts synergistically with secondary or concurrent infections, exacerbating clinical outcomes such as stunted growth and reduced egg yield, which ultimately inflicts severe financial damage upon the poultry sector [7].

The FAdV genome encodes several non-structural proteins and three major capsid proteins. The viral capsid is constructed from three primary components: hexons, penton bases, and fiber proteins. Structurally, the hexon consists of 240 trimers organized around 12 pentons, with each penton supporting two fiber proteins [8]. Notably, serotypes 1, 4, and 10 are distinguished by the presence of two fiber proteins, whereas the majority of other serotypes exhibit only a single fiber protein [9]. In recent years, genetic recombination between different strains of FAdV-E viruses has been reported, attracting significant attention [10]. Specifically, recombination between FAdV-8a and FAdV-8b has been observed through whole-genome sequencing and phylogenetic analysis. Further, the FAdV-E strains isolated from different geographical regions exhibited different genetic recombination patterns, with the prevalence of recombinant strains showing an upward trend. For example, the recombinant FAdV AH720 isolate possesses an FAdV-8b genomic backbone complemented by an FAdV-8a fiber gene. Conversely, the GDLZ strain exhibits a reciprocal arrangement, featuring an FAdV-8a backbone with the fiber gene derived from FAdV-8b [11,12]. A single infection with FAdV-8a typically does not cause severe disease; however, when co-infected with other pathogens or different serotypes of avian adenovirus, the clinical manifestations can be significantly exacerbated, raising concerns regarding the difficulty of its prevention and control [13]. Thus, continuous monitoring of the molecular evolutionary dynamics of FAdV-E, particularly the genetic variation analysis of recombination, provides a reference for formulating effective prevention and control strategies.

This work describes the isolation and whole-genome characterization of a novel virulent FAdV-8a strain, designated HuN21, recovered from poultry flocks in Hunan Province, China, that were exhibiting clinical signs of severe inclusion body hepatitis (IBH). Compared with the previously reported traditional FAdV-8a and FAdV-8b strains, the HuN21 sequence shows obvious recombination characteristics, containing genome backbone of FAdV-8a and a fiber gene from FAdV-8b. Additionally, its pathogenicity in 28-day-old and 1-day-old SPF chickens was investigated. Pathogenicity analysis revealed that HuN21 induced 100% mortality and severe IBH in 1-day-old SPF chickens, but was not lethal to 4-week-old SPF chickens.

## 2. Materials and Methods

### 2.1. Viruses and Cells

The HuN21 viral isolate was obtained from hepatic tissue samples of commercial layers suffering from severe IBH in Hunan Province, China. Chicken Leghorn male hepatocellular (LMH) cells were propagated in Dulbecco’s Modified Eagle’s Medium/nutrient mixture Ham F-12 (DMEM/F12) medium (Sigma-Aldrich, St. Louis, MO, USA) supplemented with 10% fetal bovine serum (Sigma-Aldrich, St. Louis, MO, USA) and antibiotics (100 IU/mL penicillin, 100 μg/mL streptomycin). Cultures were maintained at 37 °C in a humidified atmosphere containing 5% CO_2_.

### 2.2. Virus Isolation, Purification, and Titration

LMH cells were utilized for the isolation of viral strains from clinical samples. Upon reaching approximately 80% confluence in 75 cm^2^ flasks, the monolayers were washed twice with PBS and inoculated with 5 mL of 5-fold diluted liver homogenates. Following a 2 h adsorption period at 37 °C, the inoculum was removed and replaced with maintenance medium supplemented with 2% Fetal Bovine Serum (FBS). Cultures were monitored daily for cytopathic effects (CPE) under standard incubation conditions. The HuN21 isolate was purified by three consecutive rounds of plaque purification on LMH cells and titrated using the 50% tissue culture infectious dose (TCID_50_) method. Following testing for exogenous pathogenic microorganisms, the purified strains were subjected to PCR identification to rule out contamination by other viruses, including infectious bursal disease virus (IBDV), fowl adenovirus 4 (FAdV-4) and chicken infectious anemia virus (CIAV).

### 2.3. Viral DNA Extraction and Sequencing

Viral DNA was extracted from the HuN21 cell culture supernatant using the BioFlux Simply P DNA/RNA Nucleic Acid Extraction Kit (BioFlux, Hangzhou City, China) according to the manufacturer’s instructions. Genomic DNA served as the template for polymerase chain reaction (PCR) amplification. The forward (F) and reverse (R) PCR primers FAdV-I F (5′-GCCACCGGAAGCTACTTTGA-3′) and FAdV-I R (5′-TTGTGATCCATGGGCATGA-3′) were designed based on the FAdV-I hexon gene and used to determine the FAdV genotype. The complete genome of the HuN21 strain was sequenced using the Illumina platform with the PE150 strategy. Qualified libraries were pooled and sequenced by Novogene Bioinformatics Technology Co., Ltd. (Beijing, China). Raw reads were quality-filtered and assembled de novo using SPAdes to obtain the whole genome.

### 2.4. Sequence Analysis

To complement the experimental samples prepared for sequencing, a comparative analysis was conducted using 18 FAdV reference isolates. These strains were selected to represent the complete spectrum of the 12 known FAdV-I serotypes, allowing for comprehensive whole-genome characterization. A phylogenetic tree was constructed using the maximum-likelihood method, and molecular analyses were conducted using MEGA11 [14]. Genome maps were constructed utilizing SnapGene 8.2.2 (GSL Biotech, Chicago, IL, USA) to illustrate the full-length genomic sequences of the isolates. To identify potential recombination events in the HuN21 genome, we performed a similarity plot analysis using SimPlot (version 3.5.1) with a sliding window of 200 bp and a step size of 20 bp [15]. Four reference sequences were selected based on the whole-genome phylogenetic analysis: FAdV-8a reference strain TR59 (KT862810), FAdV-8b reference strain HeB20 (OK188966), a known recombinant strain GDLZ (MK387061), and a local FAdV-8a/8b-related strain SDQD (OR901942).

### 2.5. Animal Experiment

Specific pathogen-free (SPF) chickens were procured from Beijing Boehringer Ingelheim Technology Co., (Beijing, China). All animal procedures were performed in strict compliance with the ethical guidelines and protocols sanctioned by the Animal Care and Use Committee of Qingdao Agricultural University (Shandong, China).

### 2.6. Pathogenicity Experiment

To evaluate the virulence of the HuN21 strain, two distinct age groups of specific pathogen-free (SPF) chickens were utilized: ten 28-day-old and sixteen 1-day-old chickens. These were intramuscularly inoculated with 10^9^ 50% tissue culture infectious dose (TCID_50_) units of HuN21 in 0.2 mL and 0.1 mL PBS, respectively. As a control group, twelve SPF chickens received PBS only and were monitored for comparative analysis.

Following inoculation, chickens were observed daily for a 5-day duration. To evaluate pathological progression, three animals from each group were euthanized at 3 and 5 days post-infection (dpi). The liver, spleen, kidneys, and bursa were collected from chickens in the 1-day-old and 28-day-old infection and negative control groups at 3 and 5 dpi. These samples were subsequently fixed, paraffin-embedded, sectioned, and processed for hematoxylin and eosin (HE) staining to identify histological lesions induced by HuN21. Real-time PCR analysis was performed on tissue samples collected from the heart, liver, spleen, lungs, kidneys, thymus and bursas of the two groups to determine the distribution of the virus across different tissue types.

### 2.7. Real-Time PCR

Real-time PCR was conducted using an Archimed X4 Series Medical Fluorescence Quantitative PCR Instrument (RocGene, Xuzhou City, China). The primers used were F 5′-AAAGGCGCGGAGCTGACGG-3′ (forward primer) and R 5′-TGGGGTCCACGTTATCGGTTTC-3′ (reverse primer). A 175 bp fragment sequence was cloned into a pUC19 vector and 10^3^ to 10^8^ copies/µL were used as the PCR template to generate a standard curve. The chicken OVO gene was used as the internal reference standard. The final concentration of the PCR amplifiers was calculated based on the copy number of FAdV-8b/OVO.

### 2.8. Statistical Analysis

Differences between two groups were evaluated by Student’s t-test using GraphPad Prism version 9.5.1 (GraphPad Software, La Jolla, CA, USA). Differences were considered statistically significant at *p* < 0.01.

## 3. Results

### 3.1. Isolation and Identification of the HuN21 Strain

**Figure 1:**
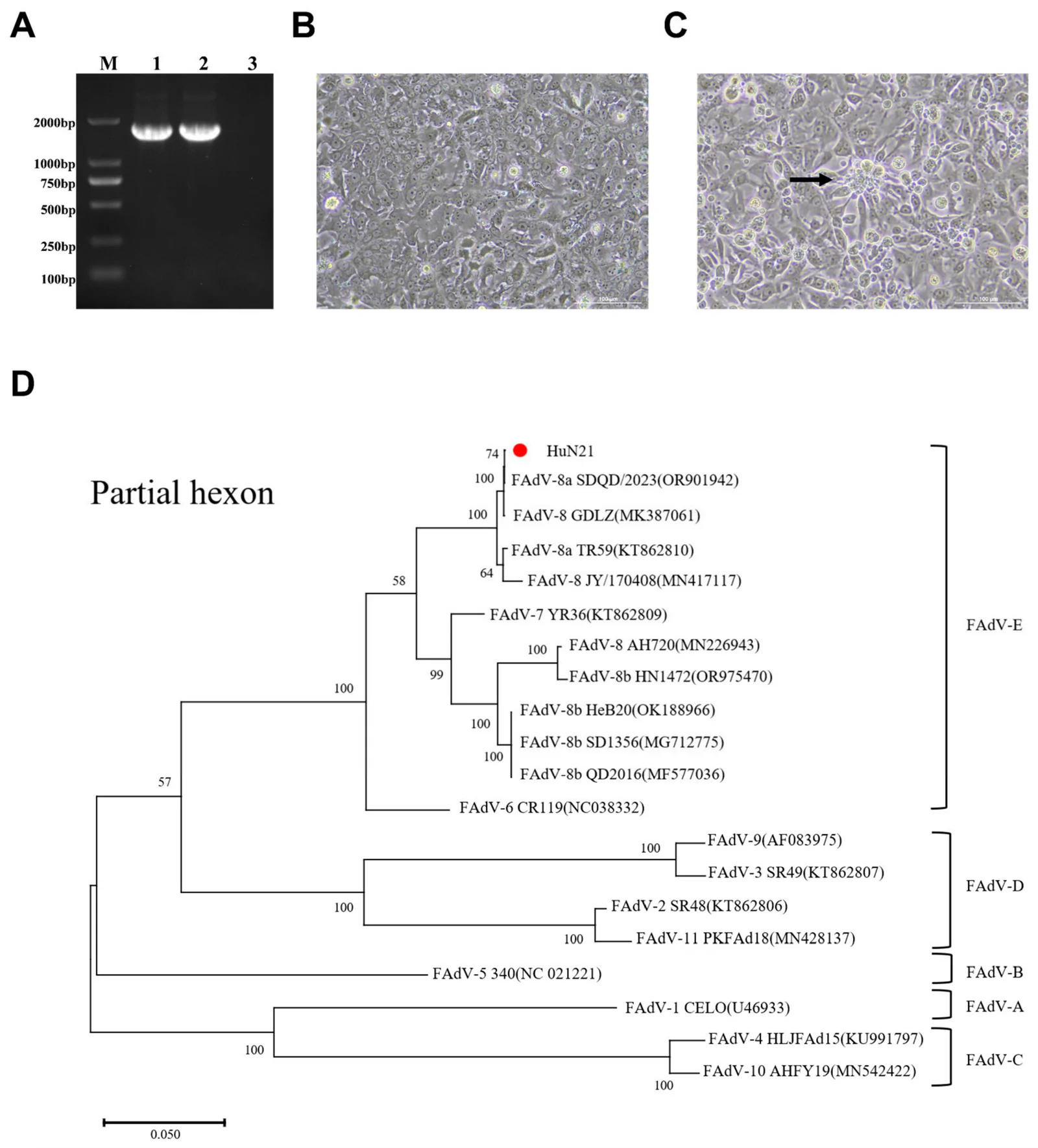
Characterization and purification of the FAdV-8a strain HuN21. (**A**) Amplification of a 1655 bp region from the hexon gene was performed using a DNA template extracted from a clinical liver specimen (Lane 1); the DNA template from purified HuN21 (Lane 2); and the DNA template from mock LMH cells (Lane 3). (**B**) Non-infected LMH cells showed no cytopathic effect (CPE). (**C**) Typical CPE of HuN21 infection in LMH cell cultures. The black arrow indicates characteristic rounding and clustering of the cells. (**D**) Phylogenetic reconstruction of the FAdV-8a isolate HuN21 using hexon gene sequences. The red dot denotes the HuN21 isolate characterized in this study. Accession numbers for the reference L1 hexon gene sequences are listed in parentheses.

Viral isolates were obtained from chickens with IBH, after which the 1655-bp region of the FAdV hexon gene was amplified by PCR (Figure 1A) and sequenced. HuN21 viruses were isolated from LMH cells, and cytopathic effects (CPE) were observed after one passage, including cell rounding and clumping (Figure 1B,C). Phylogenetic analyses of the partial hexon gene sequence confirmed that the pathogenic agent causing IBH in chickens belonged to the genus *Aviadenovirus*, species E, serotype 8a, and was designated as strain HuN21 (Figure 1D). PCR screening confirmed that the plaque-purified HuN21 stock was negative for FAdV-4, IBDV, and CIAV, ruling out the possibility of co-infection in the subsequent pathogenicity experiments.

### 3.2. Genome Organization of HuN21

**Figure 2:**
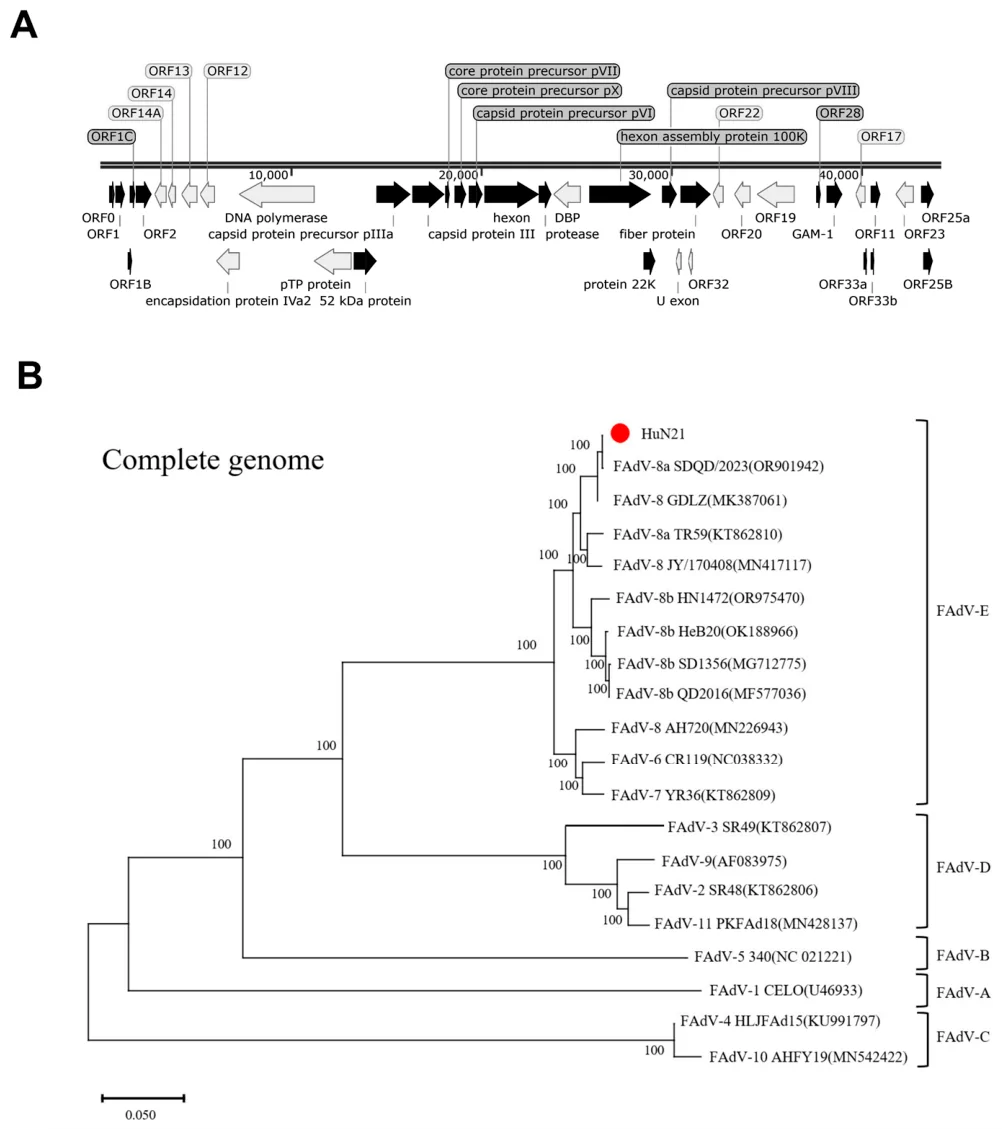
Genomic organization and evolutionary relationships of the HuN21 strain. (**A**) Diagram depicting the genomic structure of HuN21, with the locations of the 39 predicted open reading frames (ORFs). The black and white arrows indicate the positions and orientations of these ORFs. (**B**) Phylogenetic analysis of HuN21 based on whole-genome sequences. The red dot denotes the HuN21 strain characterized in this study.

The complete genome of HuN21 was determined to be 44,080 bp in length with a G + C content of 58%, similar to that of other FAdVs. Open-reading frames (ORFs) were also predicted. A schematic representation of the positions and relative sizes of all 39 ORFs that could potentially encode functional proteins is presented in Figure 2A. The GenBank accession number for the complete genome sequence of the HuN21 isolate reported in this study is PZ325483. According to the complete genome sequence phylogenetic tree analysis, HuN21 belongs to FAdV E 8a (Figure 2B). To quantitatively evaluate the genetic relationship between HuN21 and the relevant FAdV-8 strains, we performed nucleotide identity analysis based on the hexon and fiber gene regions. FAdV-8a and FAdV-8b both belong to species FAdV-E, within which nucleotide identities between different serotypes are generally high, whereas those between different species are significantly lower. The results showed that in the hexon gene region, HuN21 shared 99.2% nucleotide identity with the FAdV-8a reference strain (KT862810) and 93.1% with the FAdV-8b reference strain (OK188966), supporting its classification as serotype 8a. However, in the fiber gene region, HuN21 exhibited 99.9% identity with the FAdV-8b strain, but only 77.6% with the FAdV-8a strain. This marked difference further suggests that a recombination event may have occurred in this region. These findings are consistent with recently reported natural recombination events between FAdV-8a and FAdV-8b in China. The complete nucleotide identity data are provided in Supplementary Table S1. Novel characteristics of HuN21 viruses were identified in this study. For example, HuN21, GDLZ, and SDQD were all found to be on the same branch and are all recombinant strains of FAdV-8a and FAdV-8b, respectively. They are the most closely related and differ significantly from the other E strains.

### 3.3. Recombination Analysis of the Fiber Gene

**Figure 3:**
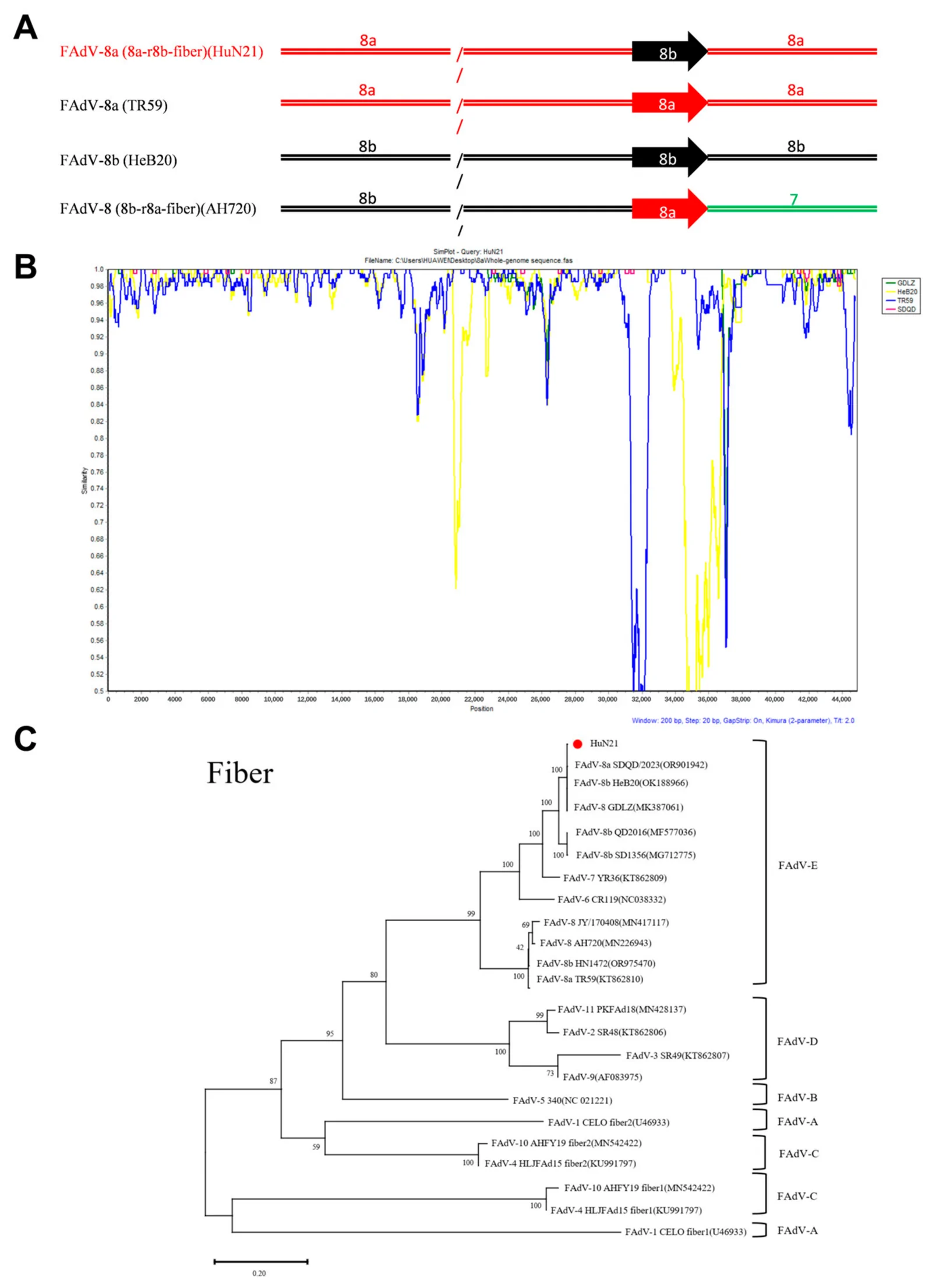
Genomic recombination and phylogenetic analysis of HuN21. (**A**) Schematic depiction of recombination events among FAdV-8a (8a-r8b-fiber; HuN21), FAdV-8a (TR59), FAdV-8b (HeB20), and FAdV-8 (8b-r8a-fiber; AH720). Arrowheads indicate the fiber gene locus. Genomic segments derived from FAdV-8a, FAdV-8b, and FAdV-7 are shown in red, black, and green, respectively. (**B**) Strains were subjected to similarity plot analysis using SimPlot version 3.5.1 with a sliding window of 200 bp and a step size of 20 bp. The HuN21 strain was used as the query sequence, and four reference sequences were selected based on whole-genome phylogenetic analysis: FAdV-8a reference strain TR59 (KT862810, blue peak plot), FAdV-8b reference strain HeB20 (OK188966, yellow peak plot), a known recombinant strain GDLZ (MK387061, green peak plot), and a local FAdV-8a/8b-related strain SDQD (OR901942, red peak plot). The *X*-axis represents the genomic position (bp), and the *Y*-axis represents the percentage of similarity to the query sequence. (**C**) Phylogenetic assessment of the major capsid fiber protein. The HuN21 strain was compared against reference sequences, with accession numbers listed in parentheses. The red dot denotes the HuN21 isolate characterized in this study.

Fiber gene recombination has been reported in recent isolates of FAdV-8a and 8b. Among recombinant strains, both HN1472 [16] and AH720 [12] contained a genome backbone originating from FAdV-8b and a *fiber* gene from FAdV-8a (Figure 3A). The SimPlot analysis revealed a clear recombination signal in the fiber gene region (Figure 3B). Specifically, in the genomic region spanning approximately 30,600 to 32,400 bp, the similarity between HuN21 and the FAdV-8a reference strain (TR59) dropped dramatically from ~99% to as low as 43–48%, while the similarity to the FAdV-8b reference strain (HeB20) remained consistently high (98–100%). Additional recombination signals were also observed in regions around 18,500–19,000 bp, 26,200–26,400 bp, and 44,200–44,600 bp, with similarity to TR59 dropping below 93% in these areas. Outside these recombinant regions, HuN21 showed high similarity (95–99%) to TR59 throughout the remainder of the genome, indicating an FAdV-8a genomic backbone. This pattern, showing high similarity to FAdV-8a in the backbone and high similarity to FAdV-8b specifically in the fiber gene region, clearly demonstrates that the fiber gene of HuN21 was acquired from FAdV-8b through natural recombination, while the rest of the genome originated from FAdV-8a. This finding is fully consistent with the phylogenetic analysis of the fiber protein (Figure 3C) and confirms that HuN21 is a recombinant strain with an FAdV-8a backbone and an FAdV-8b-derived fiber gene.

### 3.4. Pathogenicity of HuN21 in SPF Chickens

**Figure 4:**
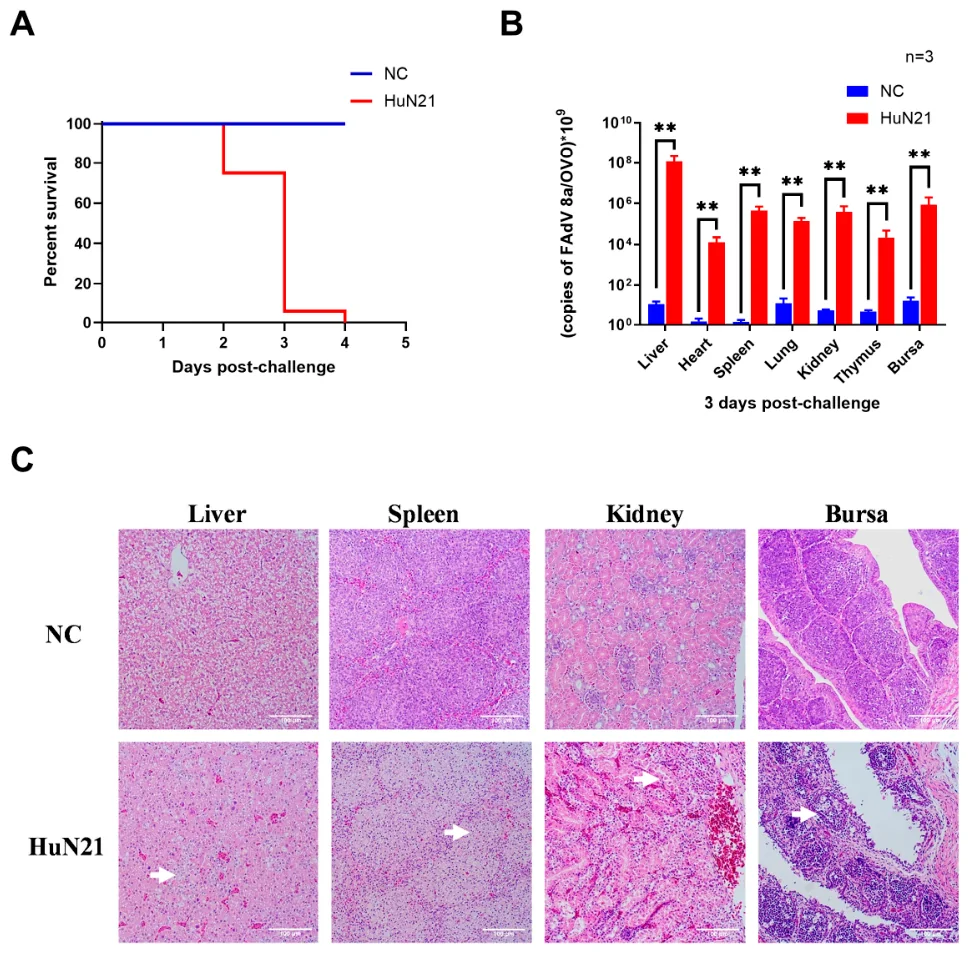
Pathogenicity of HuN21 in 1-day-old SPF chickens. (**A**) Survival rates of chickens infected with the HuN21 strain. (**B**) Virus distribution in 1-day-old chickens 3 dpi. The asterisks (**) indicate a statistically significant difference (*p* < 0.01). (**C**) Histopathological analysis of HuN21 infection in 1-day-old chickens. No evident histopathological lesions were observed in the control group. In the infected group, the livers showed focal inflammatory cell infiltration and intranuclear inclusion bodies in some hepatocytes. The spleen exhibited localized congestion, white pulp enlargement, cellular necrosis, and indistinct splenic cord structures within the red pulp. The kidneys exhibited localized congestion with marked swelling or necrosis of the renal tubular epithelial cells and narrowing of the tubular lumen. The bursa of Fabricius was shed and the medullary region was sparsely populated with cells. The white arrows highlight these representative histopathological lesions in the respective organs.

**Figure 5:**
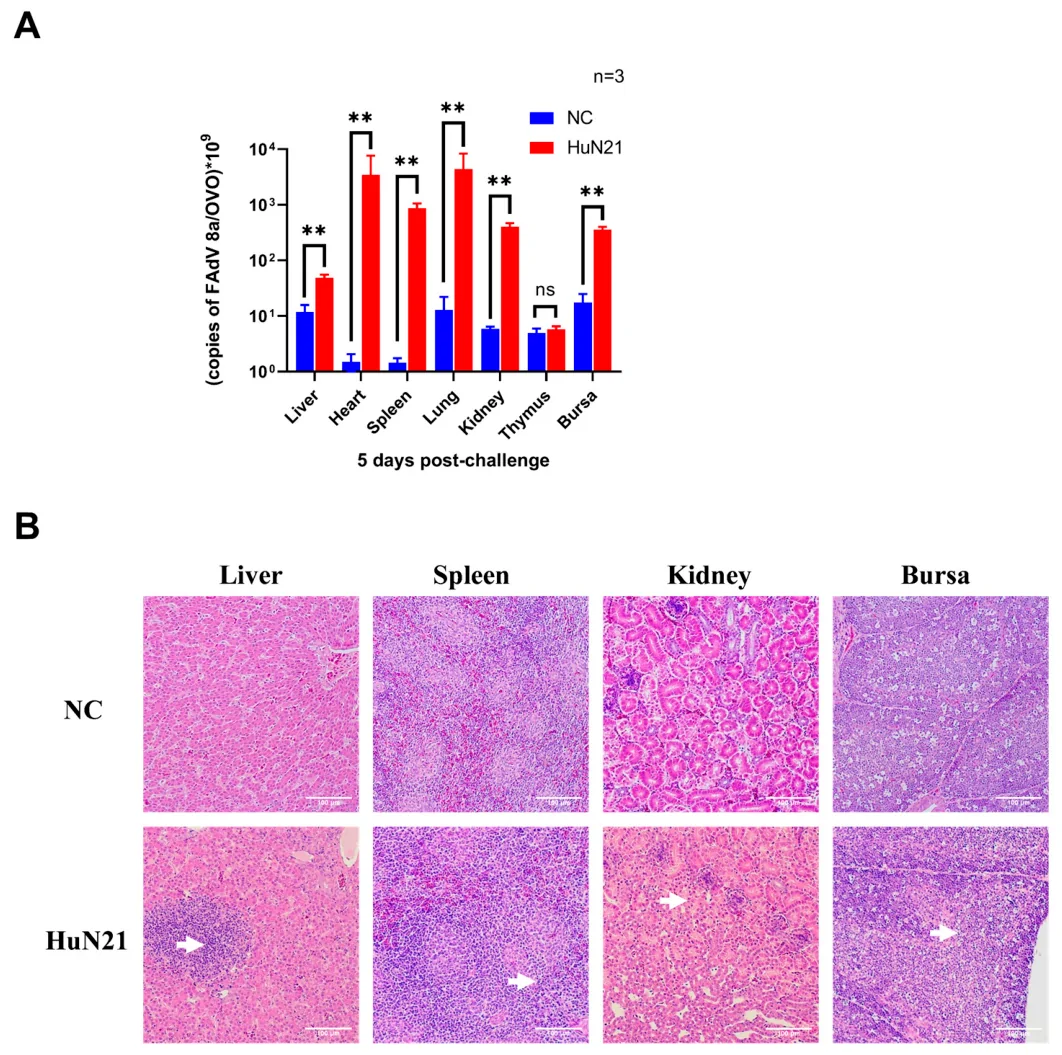
Pathogenicity of HuN21 in 28-day-old SPF chickens. (**A**) Virus distribution in 28-day-old chickens at 5 dpi. The double asterisks (**) and “ns” indicate statistically significant differences (*p* < 0.01) and no significant difference, respectively, between the HuN21-infected and NC groups. (**B**) Histopathological analysis of HuN21 infection in 28-day-old chickens. No obvious histopathological lesions were observed in the tissues of the control group. In the infected group, the liver showed extensive focal lymphocytic infiltration, with marked swelling of the hepatocytes and a small number of vacuoles in the cytoplasm of some cells. The spleen exhibited an indistinct boundary between the red and white pulp, with a marked reduction in the white pulp and unclear splenic cord structure within the red pulp. In the kidneys, glomerular swelling was observed, with necrosis in some cells and extensive focal lymphocytic infiltration in certain areas. Necrosis was also observed in some cells within the medullary region of the bursa. The white arrows highlight these representative pathological lesions in the respective organs of the infected chickens.

To evaluate the pathogenicity of HuN21 in chickens, 1- and 28-day-old SPF chickens were infected, and clinical symptoms were observed for 4 days. The clinical symptoms of infected chickens were comparable. All chickens presented with listlessness and sleepiness throughout the course of infection experiments. However, 1-day-old infected chickens showed high morbidity and mortality rates (Figure 4A), while 28-day-old infected chickens showed only clinical symptoms and no mortality. Total DNA was extracted from the tissues of infected chickens and analyzed by real-time PCR to evaluate the distribution of HuN21. At 3 days post-infection (dpi) in 1-day-old chickens, high viral loads were observed in the heart, liver, spleen, kidney, thymus, and bursa. Notably, the viral load in the liver was significantly higher than that in other tissues (Figure 4B). At 5 days post infection (dpi) in 28-day-old chickens, the viral loads in all examined tissues (including liver, spleen, kidney, lung, heart, and bursa) from the HuN21-infected group were significantly higher than those in the NC (negative control) group (Figure 5A).

### 3.5. Histopathological Analysis

Histopathological examination revealed that HuN21 infection induced distinct lesions in multiple organs of both 1-day-old and 28-day-old SPF chickens, with significantly greater severity observed in the younger age group. In 1-day-old chickens, typical IBH lesions, including intranuclear inclusion bodies in hepatocytes, extensive necrosis, and inflammatory cell infiltration, were observed in the liver, spleen, kidneys, and bursa of Fabricius (Figure 4C). In contrast, 28-day-old chickens exhibited milder pathological changes, characterized by focal lymphocytic infiltration and cellular swelling without typical inclusion bodies (Figure 5B). These findings demonstrate that the pathogenicity of HuN21 is age-dependent, with 1-day-old chickens being far more susceptible to severe tissue damage than 28-day-old chickens.

## 4. Discussion

Since 2012, the clinical incidence of IBH has followed an increasing trend in multiple regions across China, causing significant economic losses to the poultry industry [17]. Reports have indicated that this disease is associated with multiple serotypes, including 8a, 8b, and 11 [16]. In 2021, severe IBH emerged in chicken farms in Hunan Province, China. In the current study, we successfully isolated a new serotype of FAdV-8a (HuN21) from chicken livers with severe IBH during this outbreak. This study focuses on the isolation of recombinant FAdV-8a HuN21 strains from IBH outbreaks and their clinical relevance. Specifically, we conducted a comprehensive analysis of the viral genome structure and its evolutionary relationship to known FAdV strains, alongside an assessment of pathogenic potential in SPF chicken models. The phylogenetic relationships of the new isolate were determined based on the serotype-specific sequence of the hexon gene. Furthermore, Sequencing of the complete HuN21 genome was performed, and the results were submitted to GenBank (accession number PZ325483). The full-length genome sequence of FAdV-8a reported in this study provides critical insights into its genetic makeup, phylogenetic relationships, and evolutionary dynamics within the fowl adenovirus genus.

Based on comprehensive genomic characterization, the HuN21 strain was classified under species E of the FAdV-8a group. Comparative analysis demonstrated that this isolate shares a high degree of genetic similarity with reference strains across both the complete genome and the hexon gene. Phylogenetic analysis of the fiber protein indicated that this newly isolated strain is a recombinant strain resulting from a recombination event between FAdV-8a and FAdV-8b. Interestingly, the major recombination regions and genes identified in HuN21 are consistent with the recombination chimeric pattern previously reported by Chen et al. [11]. Notably, analysis revealed that the nucleotide sequence diversity within the fiber gene surpassed the variation detected in the hexon gene (such as in strains HN1472, AHT20, and GDLZ) [11,12,16], further demonstrating that such recombination events are widespread. The recombinant region encoding the fiber protein, which is responsible for binding to host cell receptors, exhibits variations that could significantly affect viral infectivity and tissue tropism [18]. Such recombination events not only increase the genetic diversity of FAdV but may further enhance its pathogenic potential. For example, HN1472 caused a mortality rate as high as 80% in SPF chickens, which was significantly higher than the 5% mortality rate observed with CH/GDLZ/201801 in prior studies, indicating that recombination may substantially enhance viral virulence under certain conditions. This finding is consistent with the previously reported notion that mutations in the FAdV genome may enhance the pathogenicity of these strains [12,19,20]. The multi-region recombination pattern observed in HuN21 is more complex than that reported for the GDLZ strain, which exhibits a relatively restricted recombination pattern largely confined to the fiber gene. This complexity may partially explain the substantial difference in pathogenicity between the two strains, with HuN21 inducing 100% mortality in 1-day-old SPF chickens compared to only 5% mortality caused by GDLZ. However, the specific contribution of each recombinant region to the enhanced virulence remains to be determined. Therefore, further research is needed to elucidate the differences in molecular mechanisms between highly virulent and non-virulent strains of FAdV-8. The FAdV-8a/8b recombination phenomenon revealed in the present study underscores the importance of continuous genomic surveillance of avian adenoviruses and the urgent need to develop new FAdV vaccines.

Herein, we further evaluated the pathogenicity of HuN21 in SPF chickens, observing varying clinical manifestations across different age groups. Pathogenicity analysis revealed that one-day-old SPF chickens exhibited high morbidity and mortality following infection, whereas four-week-old SPF chickens showed signs of illness, but no mortality. All experimental chickens infected with HuN21 exhibited moderate-to-severe clinical symptoms, while severe IBH was observed in the swollen livers of infected chickens, confirming that HuN21 could induce severe IBH. The HuN21 strain, characterized by typical IBH symptoms, exhibited remarkably high virulence in 1-day-old SPF chickens. When administered to SPF chicks via intramuscular injection, it caused significant mortality, with a mortality rate of 100%. The distribution of the virus in various tissues showed that, for chickens infected at 1 d of age, a higher viral load was observed in the liver at 3 dpi, suggesting that this may be the best tissue and time point for FAdV-8a isolation. Since we observed that HuN21 caused severe IBH, we further evaluated the histopathological changes in immune organs, finding that HuN21 significantly affected immune organs. The animal model experiments provided in this study offer useful insights and powerful tools for the development of FAdV-8a vaccines and for an in-depth investigation of the pathogenic mechanisms of FAdV-8a.

Collectively, these results revealed significant differences in pathogenicity between one-day-old and 28-day-old chickens following HuN21 infection, with younger chickens showing lethal susceptibility, and older chickens exhibiting non-lethal infection with active viral replication. This age-dependent pathogenic profile provides a valuable reference for the establishment of animal models for vaccine research. Specifically, the one-day-old chicken model, characterized by 100% mortality and high liver viral loads, could serve as a model for evaluating vaccine efficacy under severe conditions, whereas the 28-day-old model, characterized by non-lethal infection with liver viral replication, may be more suitable for assessing vaccine immunogenicity and protection against tissue damage. As such, our findings offer a practical framework for the selection of appropriate age-dependent animal models for future FAdV vaccine development.

However, this study has a number of limitations that should be borne in mind when interpreting the results. Firstly, as no direct comparison was made with the parental FAdV-8a and FAdV-8b strains, it is not yet possible to confirm whether the recombination event directly led to increased virulence. Secondly, this study did not explore in depth the specific mechanisms by which fiber-gene recombination influences pathogenicity or tissue tropism. Thirdly, we employed intramuscular injection as the route of administration, which may not fully replicate the natural course of infection; histopathological analysis was primarily descriptive and lacked quantitative lesion scoring. Fourthly, the observation period (14 days) was primarily designed to compare pathogenicity between different age groups rather than to determine viral clearance kinetics. Previous studies have shown that FAdV-8a infection in chickens can cause viremia persisting for up to 51 days. Therefore, the exact clearance time of HuN21 in 28-day-old chickens remains to be elucidated. Future studies with extended observation periods and systematic viral load monitoring are warranted to address this question.

## 5. Conclusions

In conclusion, we report the first isolation and identification of a novel recombinant FAdV-8a strain, termed HuN21, which was responsible for causing acute IBH in poultry from China’s Hunan Province. Phylogenetic assessment identified the novel isolate as a recombinant derived from FAdV-8a and FAdV-8b, with recombination events occurring in the fiber gene region. Furthermore, assessment of the pathogenicity of HuN21 in 1-day-old SPF chickens revealed that HuN21 is highly pathogenic, causing 100% mortality and inducing severe IBH, with particularly high viral loads in the liver. Interestingly, in 28-day-old chickens, the highest viral load was detected in the lung rather than the liver, followed by the heart and spleen. This differs from the tissue tropism observed in 1-day-old chickens, where the liver was the primary target organ. This age-dependent difference may reflect variations in receptor distribution or immune system maturation during development. These findings underscore the emergence of a highly pathogenic recombinant FAdV-8a strain in China, highlighting the need for ongoing virus surveillance, further investigation of the virulence mechanisms, and the development of effective vaccines against evolving FAdV strains.

## Data Availability

Sequence data generated in the present study that support the findings of this study are publicly available and were deposited in GenBank/NCBI under accession number PZ325483.

## Supplementary Materials

The following supporting information can be downloaded at: https://www.mdpi.com/article/10.3390/v18080847/s1, Table S1: Pairwise nucleotide identities between the HuN21 strain and reference strains.

## Abbreviations

The following abbreviations are used in this manuscript:

bp	Base pair
CHN	China
CO_2_	Carbon dioxide
CPE	Cytopathic effect
DMEM/F12	Dulbecco’s Modified Eagle’s Medium/Nutrient Mixture F-12
DNA	Deoxyribonucleic acid
dpi	Days post-infection
FAdV	Fowl adenovirus
FBS	Fetal bovine serum
FCS	Fetal calf serum
G + C	Guanine plus cytosine content
GE	Gizzard erosion
HE	Hematoxylin and eosin
HHS	Hepatitis-hydropericardium syndrome
HVRI	Harbin Veterinary Research Institute
IBH	Inclusion body hepatitis
kb	Kilobase
LMH	Leghorn male hepatocellular
MO	Missouri (in Sigma-Aldrich address)
ORF	Open reading frame
OVO	Ovalbumin gene (chicken reference gene)
PBS	Phosphate-buffered saline
PCR	Polymerase chain reaction
qPCR	Quantitative polymerase chain reaction
SPF	Specific pathogen-free
TCID_50_	50% tissue culture infectious dose
