# Supplementary material for: Isolation, Characterization, and Pathogenicity of a Novel Recombinant Fowl Adenovirus 8a Strain in China
Source: Viruses. 2026 Aug 2;18(8):847. doi: 10.3390/v18080847 (PMC13517140; doi:10.3390/v18080847)
Supplement: Supplementary file 1 [file viruses-18-00847-s001.zip › viruses-4445224-supplementary.pdf]

Supplementary Table S1.

Pairwise nucleotide identities between the HuN21 strain and reference strains based on the hexon gene sequences.

No.	Strain	Serotype	GenBank accession	Nucleotide identity with HuN21 (%)
1	HN1472	FAdV-8b	OR975470	93.3
2	AH720	FAdV-8	MN226943	93.2
3	CR119	FAdV-6	NC038332	91.7
4	CELO	FAdV-1	U46933	76.1
5	AHFY19	FAdV-10	MN542422	73.7
6	PKFAd18	FAdV-11	MN428137	78.9
7	SR48	FAdV-2	KT862806	79.7
8	SR49	FAdV-3	KT862807	76.3
9	HLJFAd15	FAdV-4	KU991797	73.7
10	340	FAdV-5	NC 021221	78.4
11	FAdV-9	FAdV-9	AF083975	76.2
12	GDLZ	FAdV-8	MK387061	99.9
13	HeB20	FAdV-8b	OK188966	93.1
14	JY/170408	FAdV-8	MN417117	98.6
15	QD2016	FAdV-8b	MF577036	93.1
16	SD1356	FAdV-8b	MG712775	93.1
17	SDQD/2023	FAdV-8a	OR901942	100.0
18	TR59	FAdV-8a	KT862810	99.2
19	YR36	FAdV-7	KT862809	93.8

Pairwise nucleotide identities between the HuN21 strain and reference strains based on the fiber gene sequences.

No.	Strain	Serotype	GenBank accession	Nucleotide identity with HuN21 (%)
1	CELO-fiber1	FAdV-1	U46933	37.7
2	AH720	FAdV-8	MN226943	77.4
3	CR119	FAdV-6	NC038332	85.3
4	CELO-fiber2	FAdV-1	U46933	42.4
5	AHFY19-fiber1	FAdV-10	MN542422	44.0
6	AHFY19-fiber2	FAdV-10	MN542422	49.9
7	PKFAd18	FAdV-11	MN428137	59.7
8	SR48	FAdV-2	KT862806	60.3
9	SR49	FAdV-3	KT862807	58.9
10	HLJFAd15-fiber1	FAdV-4	KU991797	43.5
11	HLJFAd15-fiber1	FAdV-4	KU991797	49.7
12	340	FAdV-5	NC 021221	55.3
13	FAdV-9	FAdV-9	AF083975	59.4
14	GDLZ	FAdV-8	MK387061	99.9
15	HeB20	FAdV-8b	OK188966	99.9
16	JY/170408	FAdV-8	MN417117	78.4
17	QD2016	FAdV-8b	MF577036	96.6
18	SD1356	FAdV-8b	MG712775	96.5
19	SDQD/2023	FAdV-8a	OR901942	99.9
20	TR59	FAdV-8a	KT862810	77.6
21	YR36	FAdV-7	KT862809	91.7
22	HN1472	FAdV-8b	OR975470	77.9
